# Supplementary material for: Pharmacological Thromboprophylaxis in People with Hemophilia Experiencing Orthopedic Surgery: What Does the Literature Say in 2023?
Source: J Clin Med. 2023 Aug 26;12(17):5574. doi: 10.3390/jcm12175574 (PMC10488906; doi:10.3390/jcm12175574)
Supplement: Supplementary file 1 [file jcm-12-05574-s001.zip › jcm-2579539-supplementary.pdf]

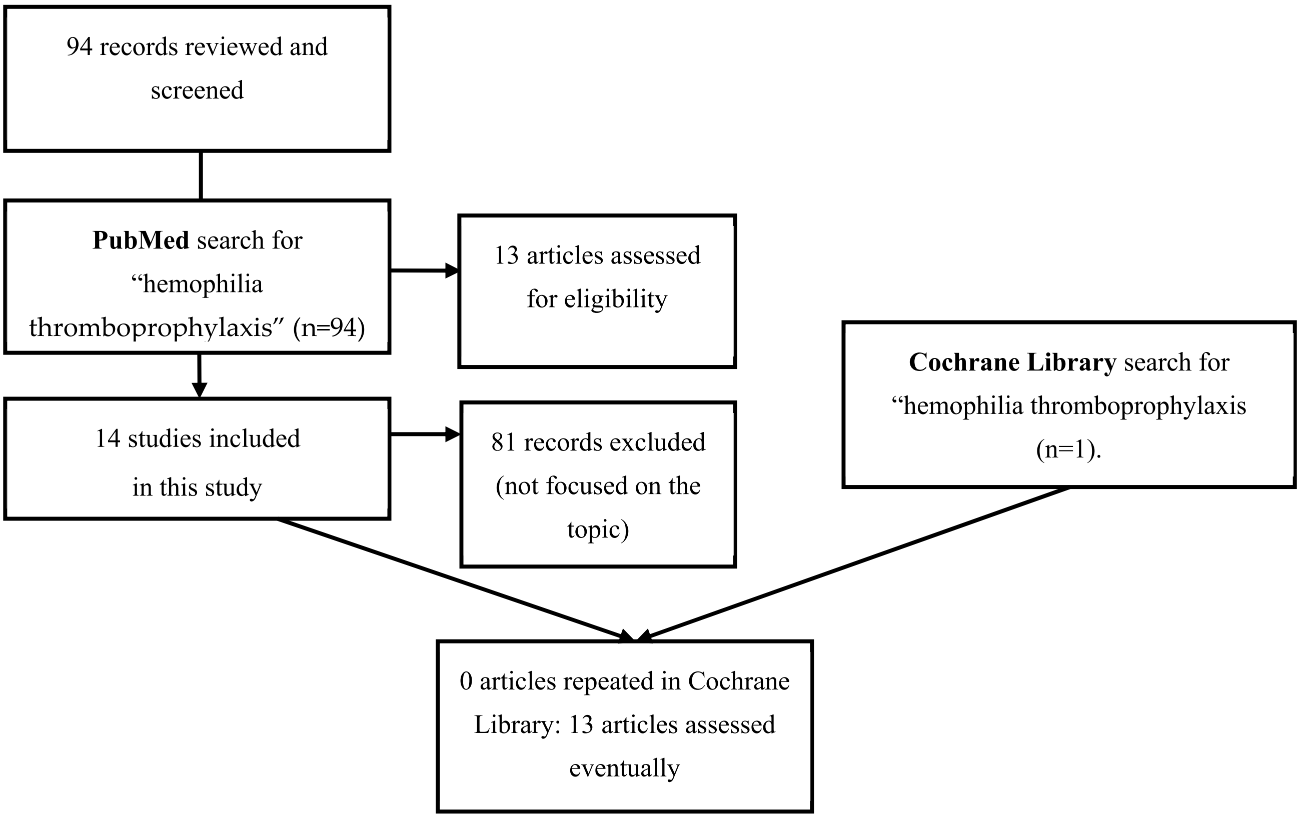

Figure S1. Flow chart of the search strategy performed regarding the role of pharmacological thromboprophylaxis after orthopedic surgery in people with hemophilia (PWH).
